# Preliminary evaluation of prototype footwear and insoles to optimise balance and gait in older people

**DOI:** 10.1186/s12877-017-0613-2

**Published:** 2017-09-11

**Authors:** Hylton B. Menz, Maria Auhl, Shannon E. Munteanu

**Affiliations:** 1 Discipline of Podiatry, School of Allied Health, College of Science, Health and Engineering, La Trobe University, Bundoora, Victoria 3086 Australia; 2 La Trobe Sport and Exercise Medicine Research Centre, School of Allied Health, College of Science, Health and Engineering, La Trobe University, Melbourne, Victoria 3086 Australia

**Keywords:** Ageing, Falls, Postural balance, Footwear

## Abstract

**Background:**

Footwear has the potential to influence balance in either a detrimental or beneficial manner, and is therefore an important consideration in relation to falls prevention. The objective of this study was to evaluate balance ability and gait patterns in older women while wearing prototype footwear and insoles designed to improve balance.

**Methods:**

Older women (*n* = 30) aged 65 – 83 years (mean 74.4, SD 5.6) performed a series of laboratory tests of balance ability (postural sway on a foam rubber mat, limits of stability and tandem walking, measured with the Neurocom® Balance Master) and gait patterns (walking speed, cadence, step length and step width at preferred speed, measured with the GAITRite® walkway) while wearing (i) flexible footwear (Dunlop Volley™), (ii) their own footwear, and (iii) prototype footwear and insoles designed to improve dynamic balance. Perceptions of the footwear were also documented using a structured questionnaire.

**Results:**

There was no difference in postural sway, limits of stability or gait patterns between the footwear conditions. However, when performing the tandem walking test, there was a significant reduction in step width and end sway when wearing the prototype footwear compared to both the flexible footwear and participants’ own footwear. Participants perceived their own footwear to be more attractive, comfortable, well-fitted and easier to put on and off compared to the prototype footwear. Despite this, most participants (*n* = 18, 60%) reported that they would consider wearing the prototype footwear to reduce their risk of falling.

**Conclusion:**

The prototype footwear and insoles used in this study improve balance when performing a tandem walk test, as evidenced by a narrower step width and decreased sway at completion of the task. However, further development of the design is required to make the footwear acceptable to older women from the perspective of aesthetics and comfort.

**Trial registration:**

Australian New Zealand Clinical Trials Registry. ACTRN12617001128381, 01/08/2017 (retrospectively registered).

## Background

Falls in older people are a major public health problem [1]. By modifying the interface between the body and the environment during weightbearing activities, footwear has the potential to influence balance in either a detrimental or beneficial manner, and is therefore an important consideration in relation to falls prevention. Several laboratory-based studies have demonstrated that elevated heels [2–4] and thick, soft soles [3–5] are detrimental to balance, while footwear with high collars [3, 6–9] and firm soles [4, 7, 8] are beneficial. Prospective studies have also shown that wearing shoes with slippery soles [10], high heels [11, 12] and reduced sole contact area [12] increase the risk of falls in older people. This is of particular concern for older women, as many styles of female footwear incorporate these potentially hazardous features.

In response to these observations, it has been suggested that the ideal safe shoe for older people at risk of falling should have a low, broad heel, a thin, firm midsole, a high collar and a textured, slip-resistant outersole [13, 14]. However, although this recommendation is a valid summary of the available literature, very few commercially available footwear styles incorporate all of these features, particularly with regard to female footwear. Furthermore, in order for such a recommendation to be widely adopted, such footwear needs to be acceptable to older people from the perspective of comfort, ease of use and aesthetics [15]. Therefore, the objectives of this study were to (i) evaluate balance ability and gait patterns in older women while wearing prototype footwear and insoles designed to improve balance, and (ii) investigate older womens’ perceptions of the footwear.

## Methods

### Participants and assessments

**Fig. 1:**
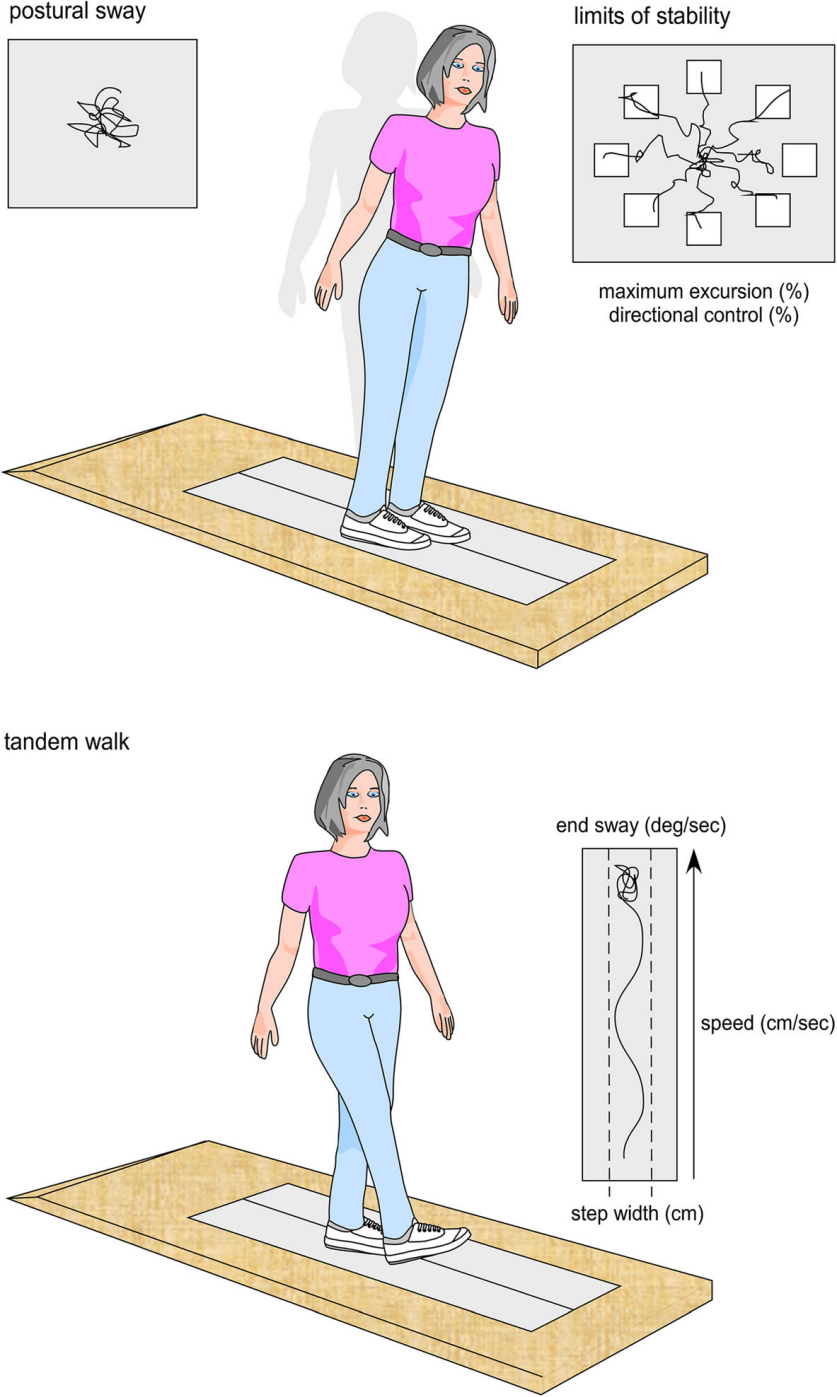
Balance testing protocol using the NeuroCom Balance Master™

This study was conducted alongside an investigation into the effects of indoor footwear on balance in community-dwelling older women [16]. These studies evaluated the same participants but laboratory testing was performed on two different occasions (one session for indoor footwear and one session for outdoor footwear). Full details of the methods, including participant eligibility, questionnaires, clinical, falls risk, balance and gait assessments and perceptions of footwear have been published [16]. Apart from the footwear conditions assessed, the only other methodological difference between the two studies was that balance testing in the current study was performed when standing on a foam rubber mat rather than on the floor. The balance testing protocol is shown in Fig. 1. Ethical approval was granted from the La Trobe University Faculty of Health Sciences Human Ethics Committee (Reference FHEC14/254), and written informed consent was obtained from all participants.

### Footwear conditions

Participants performed each of the balance and gait assessments under three footwear conditions: (i) flexible footwear, (ii) their own footwear, and (iii) the prototype footwear and insoles. For the flexible and prototype footwear, appropriate sizing was determined using the Brannock device® [17]. The order of testing was randomised to avoid order effects.

The flexible footwear (Dunlop Volley*™*, Pacific Brands, Australia) had a rubber sole of uniform 18 mm thickness, a hardness of Shore A 35 [18], and lace fixation. Across the size range, the weight of the flexible footwear was 280 – 420 g. The flexible footwear was selected as a control condition as it had no features considered to be either beneficial or detrimental to balance and could therefore be considered a ‘minimalist’ style of shoe [19].

For the ‘own footwear’ condition, participants were asked to bring to the testing session the footwear they wore most often when outdoors. The characteristics of the footwear were assessed and documented using selected items from the Footwear Assessment Tool [20].

**Fig. 2:**
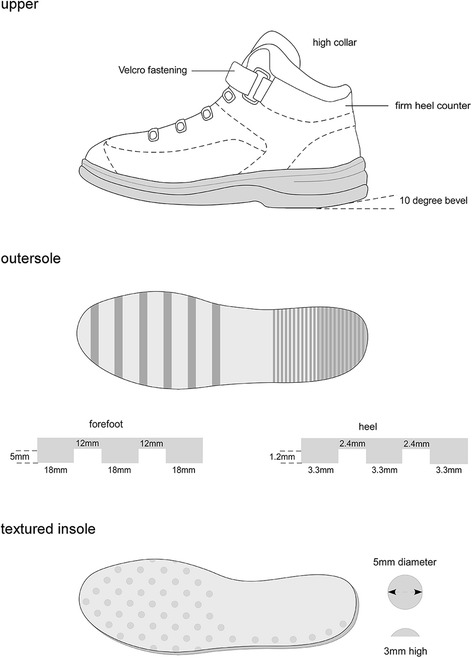
Prototype footwear and insoles. Figure reproduced with permission from *Footwear Science* 2017;9:S27–29

The prototype footwear was based on an existing model and was manufactured by Dr. Comfort® Footwear (Mequon, WI, USA). The base model (the ‘Vigor’) was selected as it incorporates many of the features previously shown to be beneficial for balance. Specifically, the footwear had a firm (Shore A hardness 55 [18]) rubber sole of 25 mm thickness under the heel and 18 mm under the forefoot, laces plus Velcro*®* fastening, a high collar to support the ankle, and a firm heel counter. Across the size range, the weight of the prototype footwear was 310 – 360 g. To create the prototype, the outersole was modified to optimise slip resistance by grinding a 10 degree bevel into the heel region [21, 22], placing grooves perpendicular to the sole (1.2 mm deep and 2.4 mm wide) across the heel surface area [23], and placing perpendicular grooves (5 mm deep and 12 mm wide) across the rest of the sole [24, 25]. A textured insole was also constructed from 4 mm thick ethyl vinyl acetate (Shore A 25 [18]) with dome-shaped projections (3 mm high and 8 mm diameter, Shore A 85 [18]) placed across the forefoot in a 15 mm diamond pattern and along the lateral border, extending to the heel. The design of the textured insole was informed by previous studies reporting improvements on balance in older people when similar insoles were worn [26, 27]. Figure 2 shows key features of the prototype footwear.

### Statistical analysis

Statistical analysis was undertaken using SPSS Version 22.0 (IBM, Armonk, NY, USA). Participants who had missing data because they were unable to complete the task were given the ‘worst’ score of the remaining sample. Differences between the three footwear conditions (flexible footwear, participants’ own footwear, and prototype footwear) were evaluated using repeated measures analysis of variance (ANOVA) with Bonferroni-adjusted post-hoc tests for pairwise comparisons. The effect sizes for all significant main effects were calculated using the eta-squared statistic (η^2^) and were interpreted using the following cut-offs: 0 – 0.06 (small), >0.06 – 0.14 (medium) and >0.14 (large) [28]. Differences in perceptions of participants’ own footwear versus the prototype footwear were evaluated using paired *t*-tests. Level of significance was set at 0.05.

## Results

### Participant characteristics

Participant characteristics are shown in Table 1, and characteristics of participants’ own footwear are shown in Table 2. Two participants had missing data for the tandem walking test (due to an inability to complete the test) and were given the ‘worst’ score of the remaining sample.

**Table 1:** Participant characteristics

Age, mean (SD) years	74.4 (5.6)
Height, mean (SD) cm	158.9 (5.77)
Weight, mean (SD) kg	75.5 (12.8)
Body mass index, mean (SD) kg/m^2^	29.9 (4.8)
Major medical conditions	
Heart disease	10 (33.3)
Diabetes	4 (13.3)
Stroke	3 (10.0)
Osteoarthritis	24 (80.0)
High blood pressure	18 (60.0)
Peripheral vascular disease	2 (6.7)
Short Form-12 Version 2	
Role – physical, mean (SD)	44.4 (9.5)
Role – mental, mean (SD)	54.2 (8.6)
Incidental and Planned Exercise Questionnaire total, mean (SD) hours/week	19.8 (14.8)
QuickScreen falls risk factors	
At least one falls risk factor	27 (90.0)
Fallen in past 12 months	7 (23.3)
Use of 4 or more medications	16 (53.3)
Use of psychotropic medications	16 (53.3)
Impaired visual acuity	15 (50.0)
Impaired peripheral sensation	6 (20.0)
Failed near tandem stance test	9 (30.0)
Failed alternate step test	12 (40.0)
Failed sit-to-stand test	10 (33.3)
Total falls risk score, mean (SD)^a^	3.3 (3.0)
Falls Efficacy Scale International, mean (SD)^b^	25.2 (7.4)
Foot problems	
Hallux valgus	14 (46.7)
Lesser toe deformity	20 (66.7)
Plantar keratotic lesions	20 (66.7)
Keratotic lesions on toes	12 (40.0)
Manchester Foot Pain and Disability Index	
Pain subscale, mean (SD)^c^	2.8 (2.6)
Functional limitation subscale, mean (SD)^d^	4.7 (4.0)

Values are *n* (%) unless otherwise stated

^a^score ranges from 1 to 8.6; higher score indicates greater risk

^b^score ranges from 16 to 64; higher score indicates greater fear (low 16–19, moderate 20–27, high 28–64)

^c^Rasch-transformed score ranges from 0 to 10; higher score indicates greater impairment

^d^Rasch-transformed score ranges from 0 to 20; higher score indicates greater impairment

**Table 2:** Characteristics of participants’ own outdoor footwear

Shoe style	
Athletic shoe	8 (26.7)
Walking shoe	6 (20.0)
Sandal	5 (16.7)
Moccasin	4 (13.3)
Boot	4 (13.3)
Mary-Jane	2 (6.7)
High heel	1 (3.3)
Sole flexion point	
At MTPJs	18 (60)
Proximal to MTPJs	7 (23.3)
Distal to MTPJs	5 (16.7)
Heel height, mm – mean (SD), range	26 (9), 10–44
Sole thickness, mm – mean (SD), range	13 (6), 3–23
Sole hardness, Shore A – mean (SD), range	60 (19), 30–96
Weight, gm – mean (SD), range	265 (65), 130–359

Values are *n* (%) unless otherwise stated

### Effects of footwear on balance

Results of the repeated measures ANOVAs for the balance tests are shown in Table 3. There was no overall effect of footwear on postural sway (F = 2.6, *P* = 0.096). For the limits of stability test, there was no overall effect of footwear on maximum excursion (F = 0.5, *P* = 0.594) or directional control (F = 1.7, *P* = 0.206). For the tandem walk test, there was no overall effect of footwear on speed (F = 2.7, *P* = 0.086). However, there was a significant overall effect of footwear on step width (F = 9.3, *P* = 0.001, η^2^ = 0.40, large effect size), with post-hoc comparisons indicating that step width was significantly greater in the flexible footwear and own footwear compared to wearing the prototype footwear. There was also a significant overall effect of footwear on end sway (F = 5.6, *P* = 0.009; η^2^ = 0.29, large effect size), with post-hoc comparisons indicating that end sway was significantly greater in the flexible footwear and own footwear compared to wearing the prototype footwear.

**Table 3:** Differences in balance and gait patterns between the footwear conditions

	Flexible footwear	Own footwear	Prototype footwear	*P* value^e^
Balance				
Postural sway velocity, °/sec^c^	0.53 (0.19)	0.60 (0.26)	0.57 (0.21)	0.096
Limits of stability test				
Maximum excursion (% LOS)^b^	70.3 (15.6)	69.9 (17.4)	71.5 (15.7)	0.594
Directional control (%)^d^	57.4 (15.5)	54.0 (17.2)	55.1 (15.3)	0.206
Tandem walk test				
Speed, cm/sec^d^	17.8 (8.7)	19.4 (10.0)	18.3 (10.6)	0.086
Step width, cm^c^	11.6 (5.7)	10.2 (6.2)	7.4 (3.1)^a,b^	0.001
End sway, °/sec^c^	5.9 (3.4)	4.9 (2.0)	4.1 (2.0)^a,b^	0.009
Gait patterns				
Walking speed, cm/sec^d^	107.4 (18.1)	109.5 (19.2)	108.0 (17.8)	0.204
Cadence, steps/min^c^	110.8 (10.1)	112.0 (11.2)	111.1 (10.2)	0.173
Step length, cm^d^	58.0 (7.1)	58.5 (7.2)	58.2 (6.8)	0.544
Step width, cm^c^	59.1 (6.6)	59.6 (7.0)	59.4 (6.6)	0.303

Values are mean (SD)

^a^significantly different to flexible shoe

^b^significantly different to own shoes

*LOS* limits of stability

^c^lower scores represent better performance

^d^higher scores represent better performance

^e^ *P* value for main effect of one-way ANOVA

### Effects of footwear on gait patterns

Results of the repeated measures ANOVAs for gait patterns are shown in Table 3. There was no significant overall effect of footwear on walking speed (F = 1.7, *P* = 0.204), cadence (F = 1.9, *P* = 0.173), step length (F = 0.6, *P* = 0.544) or step width (F = 1.2, *P* = 0.303).

### Perceptions of footwear

Participants’ perceptions of their own footwear and the prototype footwear are shown in Table 4. Participants perceived their own footwear to be more attractive, comfortable, well-fitted and easier to put on and off compared to the prototype footwear, but there was no difference in perceived heaviness. When asked if they would consider wearing the prototype footwear to reduce their risk of falling, 18 (60%) said yes, 3 (10%) said no, and 9 (30%) said maybe. Of those who said no or maybe, appearance was the most commonly reported concern (*n* = 6, 50%).

**Table 4:** Differences in perceptions of own footwear and prototype footwear

	Own footwear	Prototype footwear
Attractiveness to self	62.9 (22.4)	44.0 (24.8)*
Attractiveness to others	57.1 (21.3)	37.1 (22.0)*
Comfort	84.2 (10.5)	64.0 (17.4)*
Fit	87.3 (7.0)	74.6 (14.9)*
Ease of donning and doffing	84.4 (14.7)	62.5 (20.9)*
Heaviness	30.1 (23.9)	39.5 (21.8)

Values are mean (SD) mm from 100 mm visual analog scales. Higher scores represent greater perceived attractiveness, comfort, fit, ease of donning and doffing and heaviness

*significant difference at *P* < 0.01

## Discussion

The primary objective of this study was to evaluate balance and gait patterns in older women while wearing three types of outdoor footwear: (i) flexible ‘control’ footwear, (ii) their own footwear, and (iii) prototype footwear and insoles designed to improve balance. Our findings indicate that performances on tests of postural sway and limits of stability did not differ between the three footwear conditions, nor were there any differences in temporo-spatial gait parameters (walking speed, cadence, step length or step width). However, balance performance when undertaking the tandem walking test was enhanced while wearing the prototype footwear, as evidenced by reductions in step width and postural sway at the completion of the test. These findings suggest that the prototype footwear may improve lateral stability in older women.

There are two main explanations for the lack of differences in postural sway, limits of stability and gait patterns between the footwear conditions. Firstly, the flexible footwear we used as the control condition had no features considered to be beneficial to balance, but equally had no features that were clearly hazardous. Participants’ own footwear was also generally good, with many wearing athletic or walking footwear, which has been shown to be associated with the lowest falls risk [29]. A likely explanation for this is that most of the participants were recruited from a podiatry clinic database, where appropriate footwear would have been frequently emphasised. Secondly, these tests may not have been challenging enough to the postural control system, as the standing tests were conducted in a bipedal stance position and the walking tests were conducted on a level surface. It is possible that greater differentiation between the footwear conditions may have been obtained by testing responses to postural perturbations [30] or by conducting the walking tests on an irregular surface [31].

The observed improvement in the tandem walk test when wearing the prototype footwear, however, is a notable finding, as several studies have demonstrated the importance of lateral stability in relation to risk of falling. Older people who fall have been shown to exhibit increased lateral sway when standing in bipedal [32, 33], near-tandem [34] and unipedal [35] positions, increased lateral stepping reactions to recover balance in response to postural perturbation [36, 37], and an increased stride width when walking [38]. We found that step width when undertaking the tandem walk test was significantly narrower with the prototype footwear, which indicates that participants had less of a need for more lateral foot placement to control the lateral displacement of the centre of mass. Furthermore, postural sway was significantly reduced at the completion of the task, indicating better balance recovery in response to the lateral instability induced by tandem walking. Several features of the prototype footwear may have been responsible for this improvement, including the supportive heel collar (by providing mechanical resistance to excessive ankle movement [39] and enhanced tactile feedback of ankle position [40]), the large surface area of the sole [41, 42], and the textured insole providing tactile feedback relating to lateral displacement of the centre of mass [26, 27].

The secondary objective of this study was to investigate older womens’ perceptions of the prototype footwear, as in order to be considered a practical intervention, such footwear needs to be acceptable to older people from the perspective of comfort, ease of use and aesthetics. Perhaps not surprisingly, participants perceived their own footwear to be more attractive, comfortable, well-fitted and easier to put on and off compared to the prototype footwear. However, when asked if they would consider wearing the prototype footwear to reduce their risk of falling, 60% said yes, 10% said no, and 30% said maybe, with the appearance of the footwear being the most commonly reported concern. This finding is encouraging, as the initial prototype was designed primarily with function in mind, and little attention was given to aesthetics. For example, the prototype footwear had a black leather upper and hiking boot-style eyelets; both features that could easily be modified to improve aesthetics without impacting greatly on function.

The findings of this study need to be interpreted in the context of methodological limitations. First, participants were only provided with a brief period of time to acclimatise to the different footwear conditions before undertaking the balance tests. This is likely to have disadvantaged the prototype footwear, as the leather upper was relatively stiff, and the high collar and textured insole would have been novel to most participants. Second, because women are more likely to fall and wear potentially hazardous footwear, we specifically recruited older women into the study, so we cannot be certain that the findings are generalisable to older men. Third, it could be argued that a frailer group of older women with a higher risk of falling may have been a more appropriate target sample. However, our prototype footwear is designed to be worn outdoors, and it has been shown that older people who fall outdoors are more physically active and healthy than those who fall indoors [43]. Fourth, our assessment protocol did not include any tests specifically targeting slip resistance, so the slip resistant features of the outersole of the prototype footwear were not directly evaluated. However, the outersole design features have previously been shown to enhance slip resistance in both mechanical tests [21, 22, 24, 25] and gait studies [23]. Finally, the protocol we used does not allow us to delineate the relative contribution of the footwear and insoles to balance performance.

## Conclusion

This preliminary study has shown that the prototype footwear and insoles do not influence standing balance, leaning balance or temporo-spatial gait parameters, but improve balance when performing a tandem walk test, as evidenced by a narrower step width and decreased sway at the completion of the task. However, further research is required to evaluate the footwear under more challenging conditions (including responses to postural perturbation, and walking on irregular or slippery surfaces), and to modify the design to make the footwear acceptable to older women from the perspective of aesthetics and comfort. Finally, to determine whether wearing such footwear can contribute to the prevention of falls, a randomised trial using prospectively-documented incident falls as the primary outcome measure would need to be conducted.

## References

[1] Tinetti ME, Speechley M, Ginter SF (1988). Risk factors for falls among elderly persons living in the community. New Eng J Med.

[2] Lord SR, Bashford GM (1996). Shoe characteristics and balance in older women. J Am Geriatr Soc.

[3] Menant JC, Steele JR, Menz HB, Munro BJ, Lord SR (2008). Effects of footwear features on balance and stepping in older people. Gerontology.

[4] Menant JC, Perry SD, Steele JR, Menz HB, Munro BJ, Lord SR (2008). Effects of shoe characteristics on dynamic stability when walking on even and uneven surfaces in young and older people. Arch Phys Med Rehabil.

[5] Robbins SE, Gouw GJ, McClaran J (1992). Shoe sole thickness and hardness influence balance in older men. J Am Geriatr Soc.

[6] Lord SR, Bashford GM, Howland A, Munro B (1999). Effects of shoe collar height and sole hardness on balance in older women. J Am Geriatr Soc.

[7] Menant JC, Steele JR, Menz HB, Munro BJ, Lord SR (2009). Rapid gait termination: effects of age, walking surfaces and footwear characteristics. Gait Posture.

[8] Menant JC, Steele JR, Menz HB, Munro BJ, Lord SR (2009). Effects of walking surfaces and footwear on temporo-spatial gait parameters in young and older people. Gait Posture.

[9] Chander H, Garner JC, Wade C (2014). Impact on balance while walking in occupational footwear. Footwear Sci.

[10] Berg WP, Alessio HM, Mills EM, Tong C (1997). Circumstances and consequences of falls in independent community-dwelling older adults. Age Ageing.

[11] Gabell A, Simons MA, Nayak USL (1985). Falls in the healthy elderly: predisposing causes. Ergonomics.

[12] Tencer AF, Koepsell TD, Wolf ME, Frankenfeld CL, Buchner DM, Kukull WA, LaCroix AZ, Larson EB, Tautvydas M (2004). Biomechanical properties of shoes and risk of falls in older adults. J Am Geriatr Soc.

[13] Menant JC, Steele JR, Menz HB, Munro BJ, Lord SR (2008). Optimizing footwear for older people at risk of falls. J Rehabil Res Dev.

[14] Aboutorabi A, Bahramizadeh M, Arazpour M, Fadayevatan R, Farahmand F, Curran S, Hutchins SW (2016). A systematic review of the effect of foot orthoses and shoe characteristics on balance in healthy older subjects. Prosthetics Orthot Int.

[15] Davis A, Murphy A, Haines TP (2013). “Good for older ladies, not me”. How elderly women choose their shoes. J Am Podiatr Med Assoc.

[16] Menz HB, Auhl M, Munteanu SE (2017). Effects of indoor footwear on balance and gait patterns in community-dwelling older women. Gerontology.

[17] Menz HB, Auhl M, Ristevski S, Frescos N, Munteanu SE (2014). Evaluation of the accuracy of shoe fitting in older people using three-dimensional foot scanning. J Foot Ankle Res.

[18] American Society for Testing and Materials: D2240–97 Standard test Method for Rubber Property - Durometer Hardness. In: Annual Book of ASTM Standards*.* Philadelphia: ASTM Publishers; 1997.

[19] Esculier JF, Dubois B, Dionne CE, Leblond J, Roy JS (2015). A consensus definition and rating scale for minimalist shoes. J Foot Ankle Res.

[20] Barton CJ, Bonanno D, Menz HB (2009). Development and evaluation of a tool for the assessment of footwear characteristics. J Foot Ankle Res.

[21] Lloyd D, Stevenson MG (1989). Measurement of slip resistance of shoes on floor surfaces. Part 2: effect of a bevelled heel. J Occup Health Safety.

[22] Menz HB, Lord SR (2001). Slip resistance of casual footwear: implications for falls in older adults. Gerontology.

[23] Liu L, Lee YH, Lin CJ, Li KW, Chen CY (2013). Shoe sole tread designs and outcomes of slipping and falling on slippery floor surfaces. PLoS One.

[24] Li KW, Chen CJ (2004). The effect of shoe soling tread groove width on the coefficent of friction with different sole materials, floors and contaminants. Appl Ergon.

[25] Li KW, Wu HH, Lin YC (2006). The effect of shoe sole tread groove depth on the friction coefficient with different read groove widths, floors and contaminants. Appl Ergon.

[26] Maki BE, Perry SD, Norrie RG, McIlroy WE (1999). Effect of facilitation of sensation from plantar foot-surface boundaries on postural stabilization in young and older adults. J Gerontol.

[27] Perry SD, Radtke A, McIlroy WE, Fernie GR, Maki BE (2008). Efficacy and effectiveness of a balance-enhancing insole. J Gerontol A Biol Sci Med Sci.

[28] Cohen J (1988). Statistical power analysis for the behavioral sciences.

[29] Koepsell TD, Wolf ME, Buchner DM, Kukull WA, LaCroix AZ, Tencer AF, Frankenfeld CL, Tautvydas M, Larson EB (2004). Footwear style and risk of falls in older adults. J Am Geriatr Soc.

[30] Maki BE, McIlroy WE (2005). Change-in-support balance reactions in older persons: an emerging research area of clinical importance. Neurol Clin.

[31] Menz HB, Lord SR, Fitzpatrick RC (2003). Acceleration patterns of the head and pelvis when walking are associated with risk of falling in community-dwelling older people. J Gerontol A Biol Sci Med Sci.

[32] Maki BE, Holliday PJ, Topper AK. A prospective study of postural balance and risk of falling in an ambulatory and independent elderly population. J Gerontol. 1994;49:M72–84. 10.1093/geronj/49.2.m72 8126355

[33] Williams HG, McClenaghan BA, Dickerson J (1997). Spectral characteristics of postural control in elderly individuals. Arch Phys Med Rehabil.

[34] Lord SR, Rogers MW, Howland A, Fitzpatrick R (1999). Lateral stability, sensorimotor function and falls in older people. J Am Geriatr Soc.

[35] Crosbie WJ, Nimmo MA, Banks MA, Brownlee MG, Meldrum F (1989). Standing balance responses in two populations of elderly women: a pilot study. Arch Phys Med Rehabil.

[36] Rogers MW, Hedman LD, Johnson ME, Cain TD, Hanke TA (2001). Lateral stability during forward-induced stepping for dynamic balance recovery in young and older adults. J Gerontol A Biol Sci Med Sci.

[37] Hilliard MJ, Martinez KM, Janssen I, Edwards B, Mille ML, Zhang Y, Rogers MW (2008). Lateral balance factors predict future falls in community-living older adults. Arch Phys Med Rehabil.

[38] Maki BE (1997). Gait changes in older adults: predictors of falls or indicators of fear?. J Am Geriatr Soc.

[39] Ottaviani RA, Ashton-Miller JA, Kothari SU, Wojtys EM (1995). Basketball shoe height and maximal muscular resistance to applied ankle inversion and eversion moments. Am J Sports Med.

[40] Robbins SE, Waked E, Rappel R (1995). Ankle taping improves proprioception before and after exercise. Br J Sports Med.

[41] Hoogvliet P, Duyl WAV, Bakker JVD, Mulder PGH, Stam HJ (1997). Variations in foot breadth: effect on aspects of postural control during one-leg stance. Arch Phys Med Rehabil.

[42] Yamaguchi T, Cheng KC, McKay SM, Maki BE (2015). Footwear width and balance-recovery reactions: a new approach to improving lateral stability in older adults. Gerontechnology.

[43] Kelsey JL, Berry SD, Procter-Gray E, Quach L, Nguyen US, Li W, Kiel DP, Lipsitz LA, Hannan MT (2010). Indoor and outdoor falls in older adults are different: the maintenance of balance, independent living, intellect, and zest in the elderly of Boston study. J Am Geriatr Soc.

